# Clinical and CT angiography characteristics of Chinese stroke patients with carotid web: a retrospective study

**DOI:** 10.3389/fneur.2026.1828820

**Published:** 2026-06-17

**Authors:** Zhuoman Chen, Jingjing Cai, Xianfu Hu, Jiaming Huang, Yougang Ke, Jing Hu, Siping Luo, Lijie Ren

**Affiliations:** 1 Department of General Practice, Shenzhen Second People's Hospital, Shenzhen, Guangdong, China; 2 Department of General Practice, Shenzhen Hospital of Integrated Traditional Chinese and Western Medicine, Shenzhen, Guangdong, China; 3 Department of Neurology, Shenzhen Second People's Hospital, Shenzhen, Guangdong, China; 4 Department of Gastroenterology, The People's Hospital of Longhua, Shenzhen, Guangdong, China; 5 Department of Rheumatology and Immunology, Shenzhen Second People's Hospital, Shenzhen, Guangdong, China; 6 Department of Radiology, Shenzhen Hospital of Integrated Traditional Chinese and Western Medicine, Shenzhen, Guangdong, China; 7 Department of Radiology, Shenzhen Second People's Hospital, Shenzhen, Guangdong, China

**Keywords:** carotid web, cerebral infarction, Chinese population, computed tomography angiography, fibromuscular dysplasia, ischemic stroke

## Abstract

**Background:**

Carotid web (CaW) is a recognized risk factor for ischemic stroke, histopathologically classified as intimal fibromuscular dysplasia. However, clinical and imaging characteristics of CaW may vary across ethnic populations, and data from the Chinese population remain limited. This study aimed to investigate the clinical and computed tomography angiography (CTA) characteristics of CaW in Chinese stroke patients.

**Methods:**

We retrospectively reviewed CTA examinations of 9,220 patients at a single center in southern China from November 2005 to February 2021. Patients with CaW diagnosed on CTA were identified. Demographic data, clinical presentations, comorbidities, and CTA features including location, orientation, lumen dimensions, and stenosis severity were analyzed. Comparisons between cerebral infarction and non-cerebral infarction groups were performed using independent-sample *t*-tests and Fisher's exact test.

**Results:**

A total of 34 patients (32 males, 2 females; mean age 56.97 ± 14.75 years) with 42 CaWs were identified (prevalence 0.37%). The majority of CaWs (37/42, 88.1%) were located at the common carotid artery (CCA) bifurcation, with 5 (11.9%) at the internal carotid artery (ICA) bulb. Unilateral CaW was found in 26 patients (76.5%) and bilateral in 8 (23.5%). Cerebral infarction was the most common diagnosis (21/34, 61.8%). The lumen diameter at the CaW site was significantly different between the cerebral infarction and non-infarction groups (8.97 ± 2.34 mm vs. 6.80 ± 1.90 mm, *P* = 0.008). Hypertension was significantly more prevalent in the cerebral infarction group (76.2 vs. 23.1%, *P* = 0.004). Mild stenosis (1%−30%) accounted for 64.7% of cases per NASCET criteria.

**Conclusions:**

In the Chinese population, CaW predominantly affects males and tends to be unilateral, located at the CCA bifurcation. CaW-induced mild carotid stenosis constituted the majority of cases. CaW is an important risk factor for cryptogenic cerebral infarction. The lumen diameter at CaW sites and hypertension were significantly associated with cerebral infarction, highlighting the need for careful CTA evaluation and individualized management strategies.

## Introduction

Carotid web (CaW) is defined as a shelf-like or linear intraluminal filling defect at the posterior wall of the carotid bulb on computed tomography angiography (CTA) (1, 2). Histopathologically, CaW has been classified as intimal fibromuscular dysplasia (FMD), representing a distinct variant of cervical FMD characterized by intimal hyperplasia rather than medial fibroplasia (3, 4). Over the past decade, CaW has been increasingly recognized as an important and potentially treatable cause of recurrent ischemic stroke, particularly cryptogenic stroke in younger patients (1, 5, 6).

The incidence of cryptogenic stroke associated with CaW has been reported as 3.8 per 100,000 person-years (7). Previous studies have demonstrated that CaW predominantly affects middle-aged individuals, with a mean age of approximately 46–67 years (5, 8). Most prior studies were conducted in Western populations, where CaW has been reported to be more common in females and patients of African descent (7, 9). A recent pooled multicenter analysis by Bala et al. (10) investigated the relationship between CaW morphology on CTA and stroke risk, while Khan et al. (11) reported in-hospital recurrent stroke in carotid web patients undergoing thrombectomy. CaW is associated with a high risk of recurrent stroke or transient ischemic attack (TIA) despite antithrombotic use (1, 6).

The pathophysiology of CaW-related stroke involves alterations in luminal geometry that cause flow disturbances, blood stasis, and platelet aggregation distal to the web (12, 13). Computational fluid dynamics studies have demonstrated that subjects with CaW exhibit pro-thrombotic hemodynamic profiles compared to those with carotid atherosclerosis (13). The resultant thrombus may embolize and cause ipsilateral ischemic stroke (5, 11). Despite growing recognition, data on CaW from Chinese and broader East Asian populations remain scarce (8, 14).

This study aimed to retrospectively analyze the clinical presentations and CTA features of CaW in Chinese patients, providing insights into the demographic, clinical, and radiological characteristics of this underreported condition in the Chinese population.

## Materials and methods

### Study design and population

This retrospective study reviewed CTA examinations performed at the Second People's Hospital of Shenzhen, Guangdong Province, China, from November 2005 to February 2021. A total of 9,220 patients who underwent CTA of the cervical and cranial vessels were screened. Patients diagnosed with CaW based on CTA findings were included in the analysis. The study was approved by the Ethics Review Committee of the Futian District Chronic Disease Prevention and Control Hospital of Shenzhen (Approval No.: KJKYXLLKSSCSP20210715003).

### Diagnostic criteria for CaW

CaW was defined on CTA as a thin, membrane-like intraluminal filling defect projecting into the lumen of the carotid bulb, visible on oblique sagittal sections, with corresponding septum-like changes on axial images (1, 2, 15). Multiplane reconstruction was used to improve diagnostic performance, as recommended by Abdelhamid et al. (16). If a membrane-like filling defect was visible on oblique sagittal views but no corresponding septal structure was seen on axial images, the lesion was classified as a minor protrusion (17). To differentiate CaW from atherosclerotic plaque, the following CTA criteria were applied: CaW was identified as a thin, smooth, shelf-like intraluminal membrane projecting from the posterior wall of the carotid bifurcation, without calcification, surface irregularity, or heterogeneous density. Atherosclerotic plaques were recognized by wall thickening, high-attenuation calcification on non-contrast CT, irregular surface contour, or mixed-density composition. All ambiguous cases were reviewed in consensus by at least two senior reviewers; cases without diagnostic consensus were excluded.

### Diagnostic process

All CTA images were independently reviewed by two physicians: a neuroradiologist and a neurologist. Discordant cases were adjudicated by a senior neuroradiologist, and a diagnosis of CaW required consensus of at least two of the three reviewers. Although formal inter-rater reliability statistics (Cohen's kappa) were not prospectively calculated in this retrospective analysis, the structured three-reader consensus process was employed to maximize diagnostic reliability and minimize observer bias. The absence of a formal kappa value is acknowledged as a limitation.

### CTA imaging protocol

CTA was performed using a SOMATOM Definition 256-slice CT scanner (Siemens Healthineers, Erlangen, Germany). The scan range extended from the aortic arch to the vertex of the skull. Non-ionic iodinated contrast agent (Iopamidol, 300 mg/mL; Omnipaque, GE Healthcare, Piscataway, NJ, USA) was injected *via* the median cubital vein using a dual-barrel power injector at a flow rate of 3.0–6.0 mL/s, with approximately 50 mL of contrast agent plus 30 mL of saline flush. Scan parameters were: tube voltage 135 kV, tube current 160 mAs, slice thickness 2.0 mm, scan range 1000 mm. Scanning was triggered when contrast density reached the threshold of 160 HU in the carotid artery.

### CaW measurement and assessment

CTA images were evaluated in axial (thin section), coronal, sagittal, and oblique projection views. The intraluminal length of CaW was measured on sagittal maximum intensity projection (MIP) images perpendicular to the base of the web. CaW height and base length were measured from axial and sagittal views. Web orientation was classified as centrifugal (the shelf-like projection directed outward away from the central lumen axis, creating a post-web stagnation pocket at the vessel wall) or centripetal (the projection directed inward toward the lumen center, potentially causing greater flow obstruction). This classification follows established angioarchitectural criteria (17). Web morphology, dimensions, and other imaging characteristics were also documented. The ICA lumen diameter was measured at its widest point, and the residual lumen diameter was measured at the CaW site. Von Oiste et al. (17) have demonstrated the utility of CaW angioarchitecture for stroke risk assessment. Positive vessel wall remodeling (outward adventitial expansion compensating for intimal lesion growth) was qualitatively assessed during image review; however, a formal remodeling index was not calculated due to the absence of systematic adventitial wall segmentation. Intra-observer reproducibility for lumen measurements was verified in a random subset of re-measured cases; a formal intraclass correlation coefficient (ICC) for inter-observer agreement was not calculated, which is acknowledged as a limitation.

### Stenosis grading

ICA stenosis was calculated according to the North American Symptomatic Carotid Endarterectomy Trial (NASCET) criteria: % ICA stenosis = (1 – [narrowest ICA diameter / distal normal ICA diameter]) × 100% (18). Stenosis severity was categorized as mild (1%−30%), low-moderate (30%−50%), moderate-severe (50%−70%), and severe (70%−99%).

### Statistical analysis

The Shapiro-Wilk test was used to assess normality of continuous variables. Normally distributed continuous variables were expressed as mean ± standard deviation (SD). Independent-sample *t*-tests were used to compare continuous variables between the cerebral infarction and non-cerebral infarction groups. Fisher's exact test was used for comparison of categorical variables. A two-tailed *P* ≤ 0.05 was considered statistically significant. All statistical analyses were performed using IBM SPSS Statistics version 23.0 (IBM Corp., Armonk, NY, USA).

## Results

### Demographic and clinical characteristics

Among 9,220 patients who underwent CTA, 34 patients (0.37%) with CaW were identified, yielding a total of 42 CaW lesions. The mean age was 56.97 ± 14.75 years (range: 25–84 years; median: 58.5 years). The cohort comprised 32 males (94.1%) and 2 females (5.9%), yielding a male-to-female ratio of 16:1.

The most common initial symptoms were limb numbness and motor dysfunction (13 patients, 38.2%), followed by dizziness and headache (12 patients, 35.3%), bradykinesia (4 patients, 11.8%), dysarthria (2 patients, 5.9%), and amaurosis fugax (1 patient, 2.9%). In 2 patients (5.9%), CaW was incidentally discovered during CTA for carotid stenosis or cervical trauma.

The primary diagnoses were: cerebral infarction in 21 patients (61.8%), transient ischemic attack (TIA) in 3 (8.8%), cerebral hemorrhage in 2 (5.9%), carotid stenosis/atherosclerosis in 2 (5.9%), unexplained dizziness in 2 (5.9%), and posterior circulation ischemia, benign paroxysmal positional vertigo, tension headache, or cervical fracture in 1 patient each (2.9%).

Regarding comorbidities, hypertension was present in 19 patients (55.9%), hyperlipidemia in 16 (47.1%), smoking history in 14 (41.2%), diabetes mellitus in 9 (26.5%), and alcohol use in 5 (14.7%). Elevated homocysteine levels were observed in 6 patients (17.6%).

### CTA findings

A total of 42 CaW lesions were identified in 34 patients. The majority (37 CaWs, 88.1%) were located at the CCA bifurcation, while 5 (11.9%) were at the ICA bulb. Bilateral CaW was present in 8 patients (23.5%, 16 sites), while unilateral CaW was found in 26 patients (76.5%, 26 sites). Left-sided CaW was observed in 18 cases and right-sided in 24 cases.

Regarding web orientation, 39 CaWs (92.9%) displayed centrifugal orientation (projecting away from the lumen center), while 3 (7.1%) exhibited centripetal orientation. CaWs were most commonly observed on the lateral, medial, and posterior walls of the carotid lumen.

According to NASCET criteria, mild stenosis (1%−30%) was the most common, accounting for 22 cases (64.7%), followed by low-moderate stenosis (30%−50%) in 8 cases (23.5%) and moderate-severe stenosis (50%−70%) in 4 cases (11.8%). No cases of severe stenosis (>70%) were observed (Table 1).

**Table 1:** Distribution of ICA stenosis severity according to NASCET criteria.

Stenosis grade	Number of cases	Percentage (%)
Mild (1%−30%)	22	64.7
Low-moderate (30%−50%)	8	23.5
Moderate-severe (50%−70%)	4	11.8
Severe (70%−99%)	0	0

### Comparison between cerebral infarction and non-infarction groups

Among the 34 patients, 21 (61.8%) had cerebral infarction and 13 (38.2%) did not. The mean age in the cerebral infarction group was 58.10 ± 14.41 years (range: 25–84; median: 58 years), while that in the non-infarction group was 55.15 ± 15.69 years (range: 25–79; median: 57 years). There was no significant difference in age between the two groups (*P* = 0.580).

The lumen diameter at the CaW site was significantly larger in the cerebral infarction group compared to the non-infarction group (8.97 ± 2.34 mm vs. 6.80 ± 1.90 mm, *P* = 0.008). Hypertension was significantly more prevalent in the cerebral infarction group (76.2 vs. 23.1%, *P* = 0.004). No statistically significant differences were observed in sex, diabetes, hyperlipidemia, smoking history, drinking history, ICA lumen diameter, or ICA stenosis rate between the two groups (all *P* > 0.05; Table 2).

**Table 2:** Baseline characteristics and comparison between cerebral infarction and non-infarction groups.

Variable	All patients (*n* = 34)	Infarction (*n* = 21)	Non-infarction (*n* = 13)	*P* value
Age (years), mean ± SD	56.97 ± 14.75	58.10 ± 14.41	55.15 ± 15.69	0.580
Male sex, *n* (%)	32 (94.1)	20 (95.2)	12 (92.3)	1.000^†^
Hypertension, *n* (%)	19 (55.9)	16 (76.2)	3 (23.1)	0.004^†^
Diabetes mellitus, *n* (%)	9 (26.5)	7 (33.3)	2 (15.4)	0.427^†^
Hyperlipidemia, *n* (%)	16 (47.1)	12 (57.1)	4 (30.8)	0.172^†^
Smoking history, *n* (%)	14 (41.2)	10 (47.6)	4 (30.8)	0.477^†^
Drinking history, *n* (%)	5 (14.7)	4 (19.0)	1 (7.7)	0.627^†^
ICA lumen (mm), mean ± SD	11.23 ± 3.05	12.00 ± 3.34	10.00 ± 2.08	0.062
CaW lumen (mm), mean ± SD	8.14 ± 2.40	8.97 ± 2.34	6.80 ± 1.90	0.008
Stenosis rate (%), mean ± SD	26.07 ± 16.94	22.37 ± 18.34	32.06 ± 12.87	0.106

ICA, internal carotid artery; CaW, carotid web; SD, standard deviation

^†^Fisher's exact test. Bold P values indicate statistical significance (P < 0.05)

### Representative CTA images

**Figure 1:**
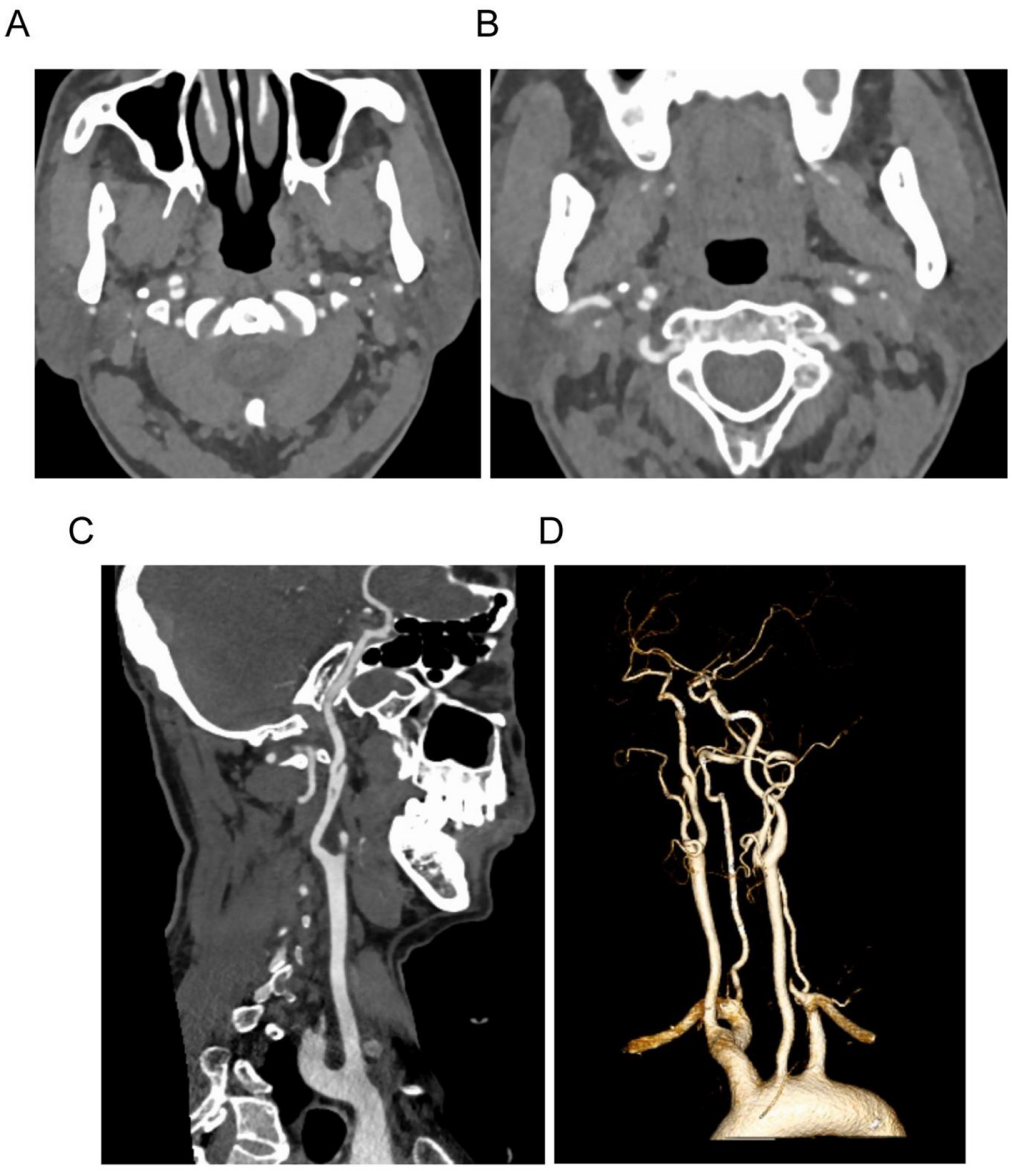
Representative CTA images demonstrating carotid web (CaW) morphology at the internal carotid artery (ICA) bulb (Patient A: 60-year-old male, non-infarction group). **(A)** Axial CTA at skull base level showing normal intracranial vasculature without significant stenosis. **(B)** Axial CTA at the carotid bifurcation level demonstrating a thin, shelf-like intraluminal filling defect (arrowhead) projecting from the posterior wall of the ICA origin, consistent with CaW. **(C)** Oblique sagittal CTA demonstrating CaW as a posterior-wall membrane-like projection (arrow) into the ICA lumen; double-headed arrow indicates the intraluminal length measurement of the web. **(D)** 3D volume-rendered CTA reconstruction of the cervical and cranial vasculature showing the overall vascular anatomy.

**Figure 2:**
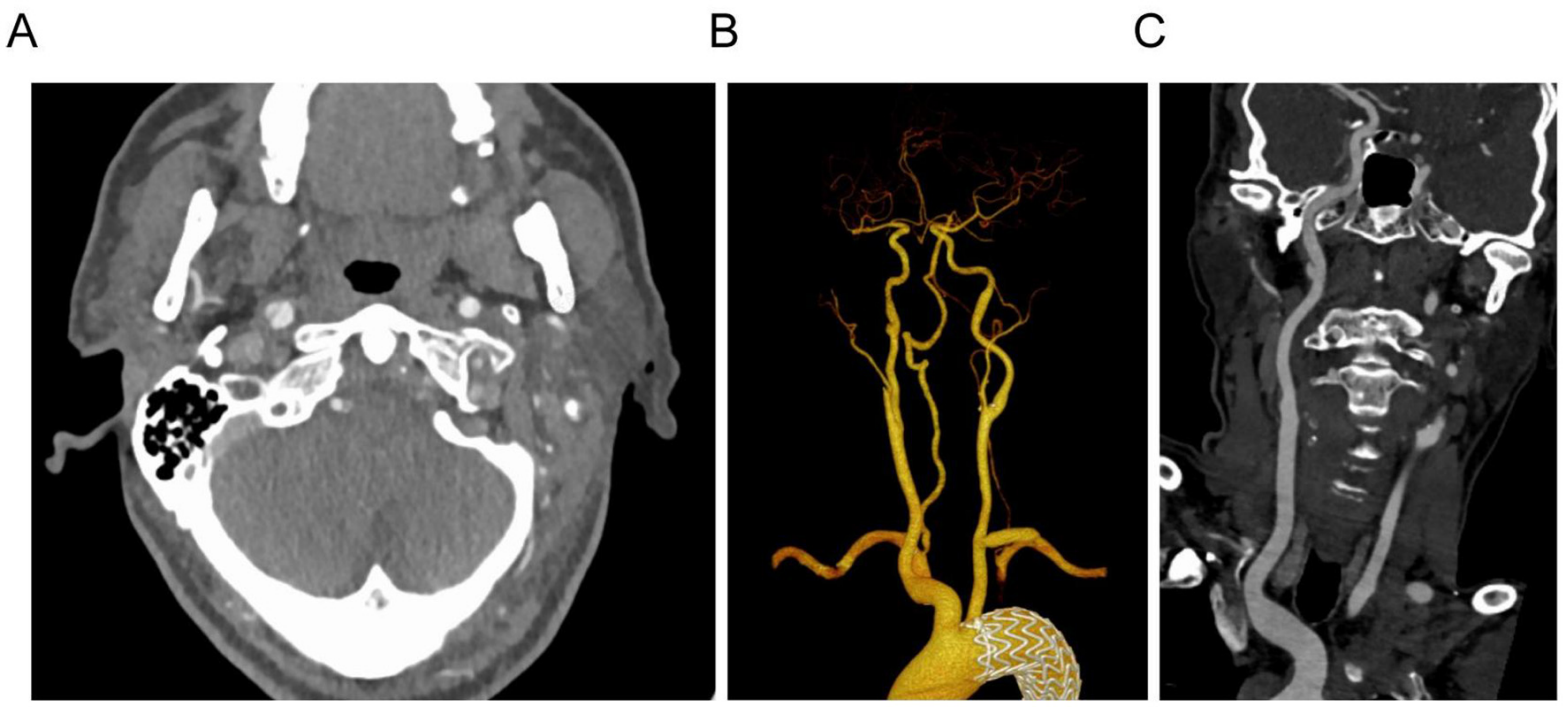
CTA images demonstrating unilateral carotid web (CaW) with centrifugal orientation at the common carotid artery (CCA) bifurcation (Patient B: 63-year-old male, non-infarction group). **(A)** Axial CTA at the carotid bifurcation level showing a thin intraluminal filling defect (arrowhead) projecting outward from the posterior wall of the ICA, consistent with centrifugal CaW morphology. **(B)** 3D volume-rendered CTA reconstruction of the cervical vasculature demonstrating the CaW (arrow) and associated mild luminal narrowing at the ICA origin. **(C)** Coronal CTA demonstrating the CaW at the carotid bifurcation, with the membrane-like web (arrowhead) visible as a linear intraluminal filling defect projecting from the posterior wall.

**Figure 3:**
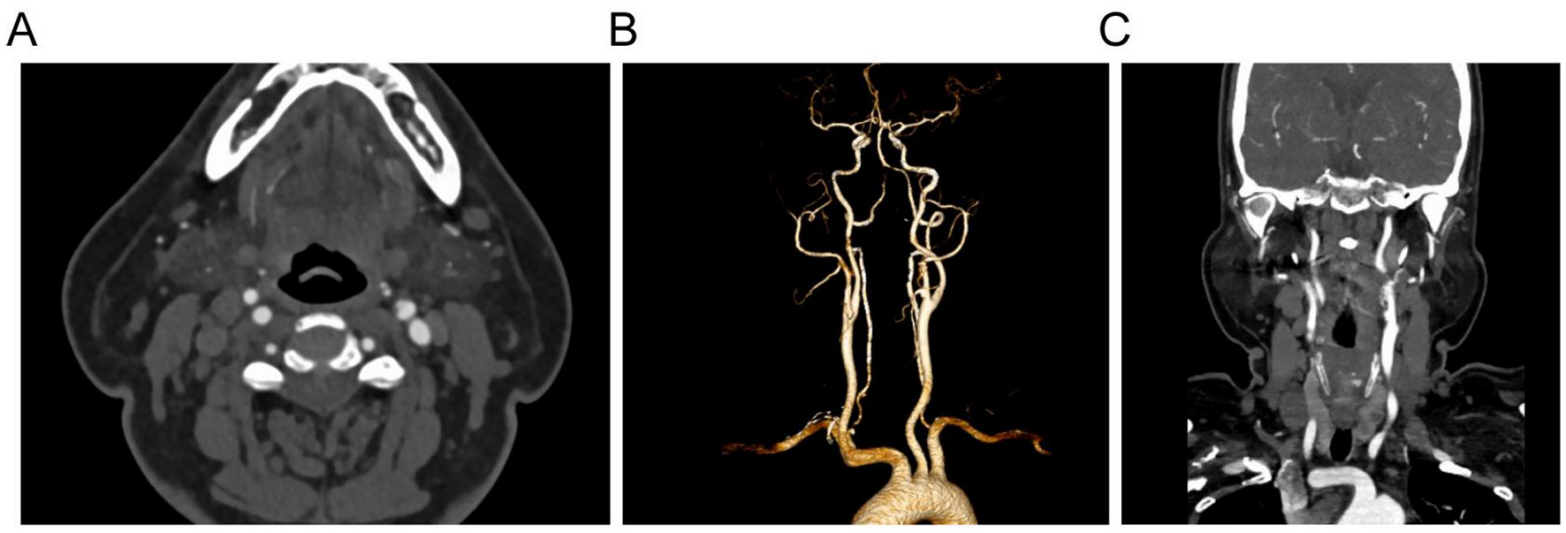
CTA images demonstrating bilateral carotid web (CaW) at the common carotid artery (CCA) bifurcation (Patient C: 63-year-old female, non-infarction group; bilateral CaW). **(A)** Axial CTA at neck level demonstrating bilateral intraluminal membrane-like filling defects (arrowheads) projecting from the posterior walls of the bilateral CCA bifurcations, consistent with bilateral CaW. The lesions are thin, smooth, and lack calcification or surface irregularity, distinguishing them from atherosclerotic plaque. Note: incidental aortic wall irregularities visible in the image represent atherosclerotic changes anatomically and morphologically distinct from the CaW lesions. **(B)** 3D volume-rendered CTA reconstruction demonstrating bilateral carotid web morphology at the bifurcation. **(C)** Coronal CTA demonstrating bilateral CaW at the carotid bifurcation with associated mild luminal narrowing bilaterally.

**Figure 4:**
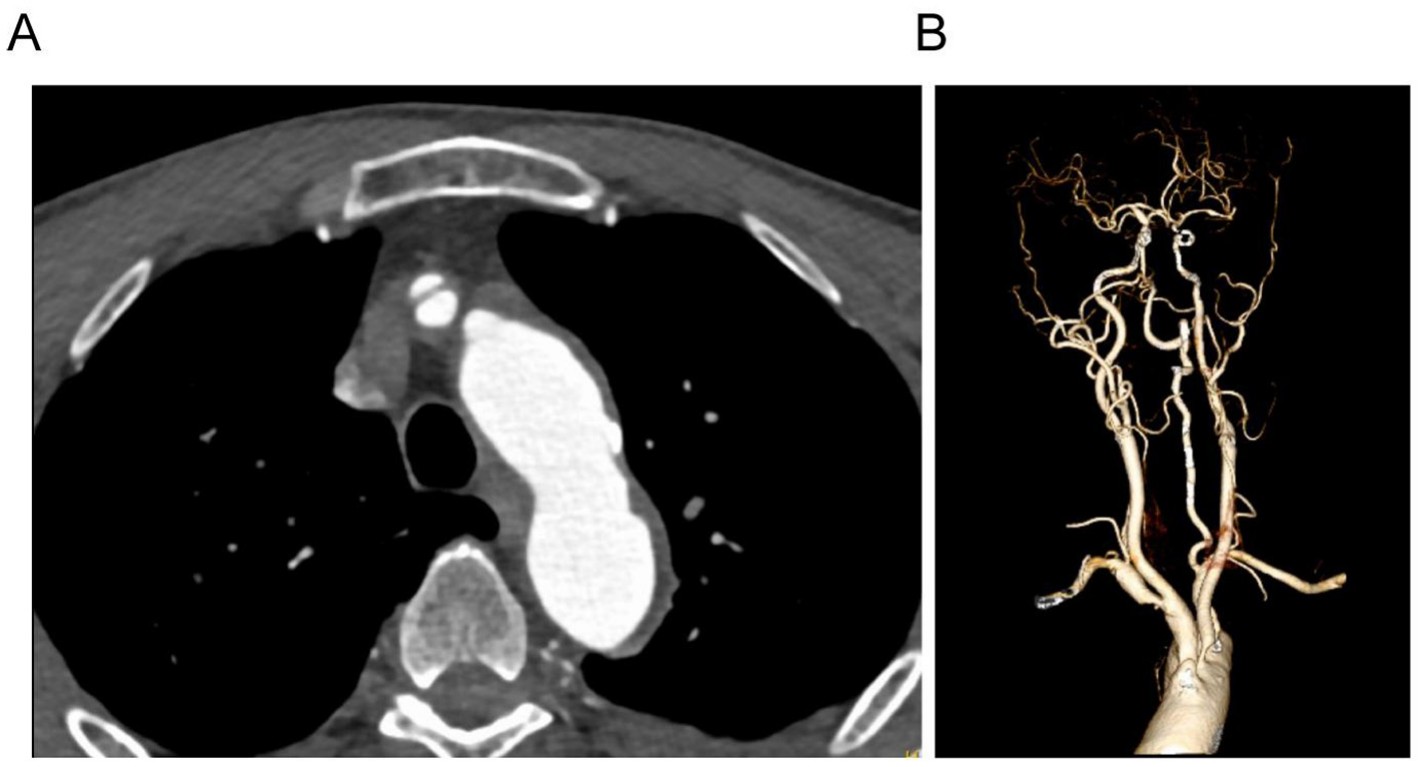
CTA images demonstrating carotid web (CaW) with associated moderate luminal stenosis (Patient D: cerebral infarction group). **(A)** Axial CTA at thoracic level showing the aortic arch and proximal great vessels at normal caliber. **(B)** 3D volume-rendered CTA reconstruction of the head and neck vasculature demonstrating CaW (arrow) at the carotid bifurcation with associated moderate-grade luminal stenosis. The web is visualized as a shelf-like posterior-wall projection creating a characteristic intraluminal irregularity at the ICA origin.

**Figure 5:**
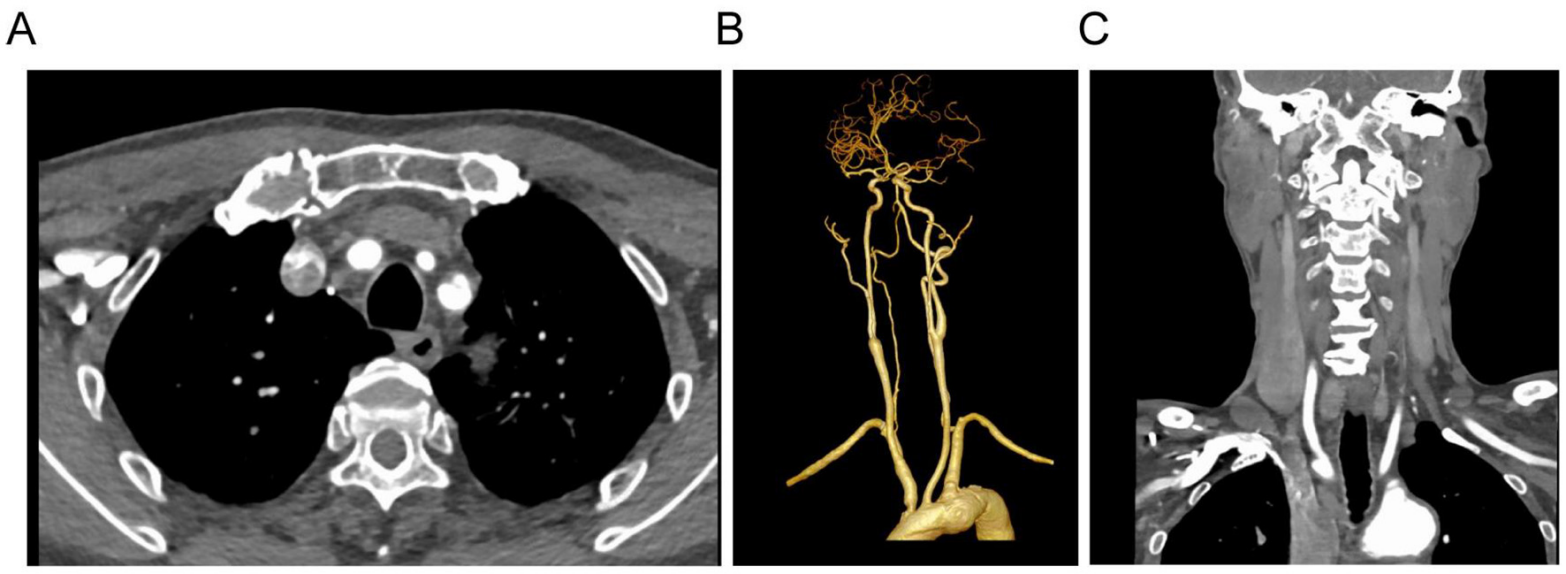
CTA images demonstrating carotid web (CaW) and associated cerebrovascular involvement in a patient with cerebral infarction (Patient E: cerebral infarction group). **(A)** Axial CTA at the thoracic inlet level showing the proximal great vessels. **(B)** 3D volume-rendered CTA reconstruction of the cervical and intracranial vasculature demonstrating CaW (arrow) at the carotid bifurcation and its spatial relationship to the downstream intracranial circulation. **(C)** Coronal CTA demonstrating CaW at the carotid bifurcation, with the web (arrowhead) visible as a linear intraluminal filling defect projecting from the posterior wall of the carotid lumen, with associated mild luminal narrowing.

Representative CTA images from five patients are presented in Figures 1–5, demonstrating the characteristic shelf-like intraluminal filling defects at the carotid bifurcation and ICA bulb region on axial, sagittal, coronal, and 3D volume-rendered reconstructions.

## Discussion

This retrospective study provides a comprehensive analysis of the clinical and CTA characteristics of CaW in a Chinese population. To our knowledge, this is one of the largest studies focusing specifically on CaW in Chinese patients, contributing to the limited but growing body of evidence on this underrecognized cause of ischemic stroke (5, 8).

In our cohort, CaW was identified in 34 out of 9,220 patients (0.37%) who underwent CTA, consistent with reported prevalence rates of 0.3%−1.0% in various populations (7, 10). Notably, the male-to-female ratio in our study was 16:1, which starkly contrasts with Western studies where CaW has been reported to be more common in females (7, 9, 18). Zhang et al. (9) demonstrated that symptomatic CaW patients were more frequently young females (67%), and most patients (70%) were of African descent. Recent case reports, such as that by Rocco et al. (18) continue to highlight the female predominance in Western populations. This difference may reflect ethnic variations in CaW epidemiology or selection bias in our study population. The pathogenesis of CaW may involve autosomal dominant familial predisposition, congenital developmental abnormalities, and possibly hormonal factors (3, 5), which could partly explain the observed sex distribution differences.

On CTA, the most common location for CaW was the CCA bifurcation (88.1%), with a smaller proportion at the ICA bulb (11.9%). This distribution is consistent with the pathological hallmark of CaW being an intimal variant of FMD that preferentially affects the carotid bulb region (3, 4). Unilateral CaW (76.5%) was significantly more common than bilateral CaW (23.5%), consistent with findings by Bala et al. (10) and Zhang et al. (9) In contrast, Haussen et al. (1) reported bilateral CaW in 58% of cases, likely due to differences in study populations and imaging protocols. Our finding underscores the importance of systematically evaluating both carotid arteries to avoid missing bilateral lesions.

The majority of CaWs (92.9%) exhibited centrifugal orientation, projecting outward from the lumen center. This morphological feature is consistent with the proposed mechanism of flow disturbance and thrombus formation, as the shelf-like projection creates a pocket of blood stasis distal to the web (1, 12, 13). Monteiro et al. (12) recently characterized the hemodynamic parameters of CaW on catheter angiography, while El Sayed et al. (13) demonstrated that subjects with CaW exhibit pro-thrombotic hemodynamics compared to those with carotid atherosclerosis.

According to NASCET criteria, the majority of CaW-associated stenosis was mild (64.7%), followed by low-moderate (23.5%) and moderate-severe (11.8%). No cases of severe stenosis (>70%) were observed. This is an important finding, as CaW typically causes only mild to moderate luminal narrowing, yet the thromboembolic potential is disproportionate to its degree of stenosis (1, 6). This paradox highlights the unique pathophysiological mechanism of CaW-related stroke, which is primarily thromboembolic rather than hemodynamic (5, 13).

A key finding of this study was the significant difference in the lumen diameter at the CaW site between the cerebral infarction and non-infarction groups (*P* = 0.008). We adopted “cerebral infarction” and “non-cerebral infarction” as group designations rather than “symptomatic” and “asymptomatic,” because the non-infarction group comprised a clinically heterogeneous population including patients with TIA, incidentally discovered CaW, unexplained dizziness, and other diagnoses—many of whom were symptomatically affected but had not sustained overt cerebral infarction. This terminology provides greater specificity for the primary clinical endpoint. Regarding large vessel occlusion (LVO) status, LVO was not systematically documented in this retrospective cohort; this is acknowledged as a limitation in the Limitations section. The cerebral infarction group had a larger CaW lumen diameter (8.97 ± 2.34 mm) compared to the non-infarction group (6.80 ± 1.90 mm). This seemingly counterintuitive finding may be explained by the fact that larger CaW structures create more significant flow disturbance and larger post-web stasis zones, facilitating thrombus formation. From a hemodynamic standpoint, a larger residual lumen diameter at the CaW level likely reflects a more prominent shelf-like protrusion, which amplifies downstream recirculation zones and prolongs the residence time of blood elements in the post-web stagnation pocket—conditions known to promote platelet activation, fibrin deposition, and *in situ* thrombus formation. El Sayed et al. (13) demonstrated using computational fluid dynamics (CFD) that CaW subjects exhibit significantly more pro-thrombotic hemodynamic profiles than those with carotid atherosclerosis, characterized by elevated oscillatory shear index and disturbed near-wall flow. Damiani Monteiro et al. (12) further confirmed pro-thrombotic flow patterns at the web site on catheter angiography. A larger absolute lumen caliber may also permit greater thrombus volume accumulation before embolization, increasing the risk of clinically significant cortical infarction. These observations underscore the inadequacy of NASCET stenosis percentage alone for CaW risk stratification and support incorporating absolute lumen diameter as a complementary risk metric in future prospective studies (12, 13, 17). Furthermore, hypertension was significantly more prevalent in the cerebral infarction group (76.2 vs. 23.1%, *P* = 0.004), confirming that hypertension is a major contributing risk factor for cerebral infarction in CaW patients.

While digital subtraction angiography (DSA) remains the gold standard for CaW diagnosis, CTA offers several advantages including non-invasiveness, rapid acquisition, high spatial resolution, and the ability to generate multiplanar reformatted images (15, 16). Abdelhamid et al. (16) demonstrated that multiplane reconstruction significantly improves the diagnostic performance of CTA in detecting carotid webs. Hou et al. (19) showed that multimodal ultrasound can differentiate CaW from CaW with plaque, while Zhu et al. (20) investigated contrast dynamics on multiphase CTA in CaW and their association with stroke. CTA remains the preferred initial imaging modality for CaW assessment (5, 15).

In our study, none of the 34 patients underwent surgical treatment for CaW. Approximately half (48.5%) received anticoagulation or antiplatelet therapy. Treatment options for CaW include anticoagulation, antiplatelet therapy, carotid endarterectomy (CEA), and carotid artery stenting (CAS) (6, 7, 21). A recent systematic review and meta-analysis by Khan et al. (6) comparing carotid revascularization vs. medical management demonstrated the superiority of revascularization for preventing recurrent stroke. Pasarikovski et al. (22) reported favorable outcomes with carotid stenting in a multicenter experience, and Bounajem et al. (23) confirmed these findings in a multi-institutional cohort study. Konieczna-Brazis et al. (24) demonstrated effective treatment with endovascular techniques, while Cao et al. (25) and Wheibe et al. (26) reported positive results with surgical management. The nationwide survey by Khan et al. (27) revealed variations in management preferences between vascular neurologists and neurointerventionalists. Fujiwara et al. (21) and Talathi and Lipsitz (28) have recently summarized current evidence and future directions for treatment.

This study has several limitations. First, it is a retrospective single-center study from a single institution in southern China, which may introduce selection and referral bias and limit generalizability to broader Chinese and East Asian populations; multicenter prospective studies are needed to validate these findings. Second, the sample size is small (*n* = 34), which precluded robust multivariate logistic regression analysis to identify independent predictors of cerebral infarction; future studies should incorporate multivariable modeling with adequate statistical power. Third, formal inter-rater reliability statistics (Cohen's kappa) were not prospectively calculated, although a structured three-reader consensus adjudication process was employed. Fourth, antithrombotic and anticoagulant treatment regimens, follow-up duration, and stroke recurrence rates were not systematically recorded, preventing evaluation of treatment efficacy and long-term prognosis. Fifth, large vessel occlusion (LVO) status and the topographic correspondence between infarct territory and the ipsilateral CaW side were not consistently documented, limiting verification of the ipsilateral thromboembolic mechanism. Sixth, histopathological confirmation of CaW was not performed; Khan et al. (4) recently provided histopathologic evidence of intimal hyperplasia in stroke-associated CaW. Seventh, formal vessel wall remodeling index quantification and ICC for lumen measurements were not performed in this retrospective study. Finally, the marked male predominance in our cohort may reflect regional referral patterns or epidemiological differences rather than the true sex-based distribution of CaW in the Chinese population.

## Conclusions

In this retrospective study of Chinese patients, CaW predominantly affected males and was mostly unilateral, located at the CCA bifurcation. Mild carotid stenosis accounted for the majority of cases. CaW is an important risk factor for cryptogenic cerebral infarction, with the lumen diameter at the CaW site and hypertension being significantly associated with cerebral infarction. Most patients were managed with medical therapy alone, suggesting that current treatment may be insufficient. Larger, multicenter prospective studies are needed to validate these findings and determine the best management strategies for CaW in the Chinese population.

## Data Availability

The original contributions presented in the study are included in the article/supplementary material, further inquiries can be directed to the corresponding author.
